# Characterization of both myocardial extracellular volume expansion and myocyte mypertrophy by CMR detect early signs of myocardial tissue remodeling in Friedreich's ataxia patients without heart failure.

**DOI:** 10.1186/1532-429X-18-S1-W7

**Published:** 2016-01-27

**Authors:** Otavio R Coelho-Filho, Ravi V Shah, Thiago D Venancio, Alberto R Martinez, Tomas G Neilan, Irene Righetti, Cynthia B da Silva, Ingrid Faber, Iscia Lopes-Cendes, Marcondes França, Michael Jerosch-Herold

**Affiliations:** 1 grid.411087.b0000000107232494Medicine, State University of Campinas - UNICAMP, Campinas, Brazil; 2 grid.38142.3c000000041936754XHarvard Medical School, Boston, MA USA

## Background

Heart Failure (HF) is the most common cause of death in Friedreich's ataxia (FRDA), a mitochondrial disease characterized by neurodegeneration, hypertrophic cardiomyopathy, caused by homozygous GAA expansions in the *FXN* gene. Recent report demonstrates that specific-gene therapy may prevent and reverse the cardiomyopathy in a mice model of FRDA. Myocardial interstitial fibrosis is a hallmark of FRDA's cardiomyopathy and a potential substrate for arrhythmias and HF. Myocardial tissue characterization by cardiac magnetic resonance (CMR) allows access to tissue-based phenotypes that may better describe LV remodeling in FRDA's cardiomyopathy.

## Methods

The aim of this study was to perform direct quantification of myocardial extracellular volume fraction (ECV) and intracellular lifetime of water (τ_ic_), a measure of cardiomyocyte hypertrophy, using T1-weighted CMR imaging in cohort of patients with FRDA without HF.

We investigated 27 FRDA patients without HF (mean age 26.8 ± 9, 12 female) and in 30 healthy volunteers as control subjects (mean age 49 ± 15) using a 3T CMR system. The T1 quantification by Look-Locker gradient-echo before and after contrast applying a 2-site model for transcytolemmal water Exchange was used for ECV and τ_ic_ quantification. Cine CMR and LGE imaging in matching locations were also performed.

## Results

FRDA patients revealed normal LVEF with increased LV Mass-index compared with health controls (for LVEF 67.3% ± 11.5 vs. 62.5% ± 6.8, P = NS; for LVMASSi 62.7 ± 23 vs. 45.1 ± 6.8 g/m^2^, p < 0.05). In 4 out 27 FRDA patients a non-ischemic LGE pattern was present. Both ECV and intracellular lifetime of water (τ_ic_) were significantly higher FRDA patients (ECV: 0.36 ± 0.04 vs. 0.28 ± 0.03, p < 0.0001; τ_ic_: 0.12 ± 0.08 vs. 0.08 ± 0.03, p < 0.005).

## Conclusions

ECV and intracellular lifetime of water (τ_ic_) determined by T1 measurements characterized early signs of myocardial tissue remodeling in FRDA with normal LVEF. Early changes in tissue-phenotypes are detectable by novel-CMR methods in FRDA patients, and may be useful to track effects of new genetic therapies for FRDA cardiomyopathy.

